# Treatment with an SLC12A1 antagonist inhibits tumorigenesis in a subset of hepatocellular carcinomas

**DOI:** 10.18632/oncotarget.10670

**Published:** 2016-07-18

**Authors:** Fei Teng, Meng Guo, Fang Liu, Ce Wang, Jiayong Dong, Lei Zhang, You Zou, Rui Chen, Keyan Sun, Hong Fu, Zhiren Fu, Wenyuan Guo, Guoshan Ding

**Affiliations:** ^1^ Department of Liver Surgery and Organ Transplantation, Changzheng Hospital, Second Military Medical University, Shanghai 200003, China; ^2^ Department of Orthopaedics, Changzheng Hospital, Second Military Medical University, Shanghai 200003, China

**Keywords:** HCC, liver cancer, SLC12A1, cancer outlier profile analysis, Bumetanide

## Abstract

A central aim in cancer research is to identify genes with altered expression patterns in tumor specimens and their potential role in tumorigenesis. Most types of tumors, including hepatocellular carcinoma (HCC), are heterogeneous in terms of genotype and phenotype. Thus, traditional analytical methods like the t-test fail to identify all oncogenes from expression profiles. In this study, we performed a meta-Cancer Outlier Profile Analysis (meta-COPA) across six microarray datasets for HCC from the GEO database. We found that gene SLC12A1 was overexpressed in the Hep3B cell line, compared with five other HCC cell lines and L02 cells. We also found that the upregulation of SLC12A1 was mediated by histone methylation within its promoter region, and that SLC12A1 is a positive regulator of the WNK1/ERK5 pathway. Consistent with *in vitro* results, treatment with the SLC12A1 antagonist Bumetanide delayed tumor formation and reduced Hep3B cell tumor size in mouse xenografts. In summary, our research reveals a novel subset of HCCs that are sensitive to SLC12A1 antagonist treatment, thereby offering a new strategy for precision HCC treatment.

## INTRODUCTION

Hepatocellular carcinoma(HCC) is the sixth most common cancer worldwide and among the leading causes of cancer-related deaths in China [1–3]. Although the understanding of HCC pathogenesis and clinical practices to treat HCC have improved [4], more studies and novel treatments are needed to improve the prognosis of patients.

Microarray experiments constitute an efficient way to identify genes relevant to cancer development. Large-scale tumor associated microarray data have been collected through decades and are available on several databases like GEO. A comparison of two sets of samples from different phenotypes (e.g., tumor and adjacent tissue) using any of several proposed methodologies [5, 6] can reveal genes with differential expression. T-statistics is the most common method; however, heterogeneous patterns of oncogene activation have been observed in the majority of cancer types. In such cases, traditional variance analysis fails to discover oncogenes that are expressed in a subset of cases.

Recently, MacDonald and *et al.* have proposed a new analytical method called “Cancer Outlier Profile Analysis” (COPA) [7] for detecting oncogenes that are abnormally expressed in only a subset of tumor samples. Here, we performed meta-COPA across six microarray datasets of hepatocellular carcinoma to identify differentially expressed genes. Top median-ranked gene SLC12A1, a member of the Na^+^-dependent subgroup of solute carriers [8], was overexpressed in 5%~25% of the samples we analyzed, suggesting it might be involved in HCC pathogenesis in a subset of liver cancer patients. We also tested the hypothesis that SLC12A1 can act as an oncogene in HCC by performing a series of *in vitro* and *in vivo* experiments. We also found that the overexpression of SLC12A1 was mediated by histone methylation changes within its promoter region. Importantly, SLC12A1 inhibition suppressed HCC cell proliferation. Finally, we tested whether an SLC12A1 antagonist could be used as a drug to treat HCC in nude mice xenograft models.

## RESULTS

### Analysis of six datasets by COPA method reveals that SLC12A1 is a potential oncogene in HCC

**Figure 1:**
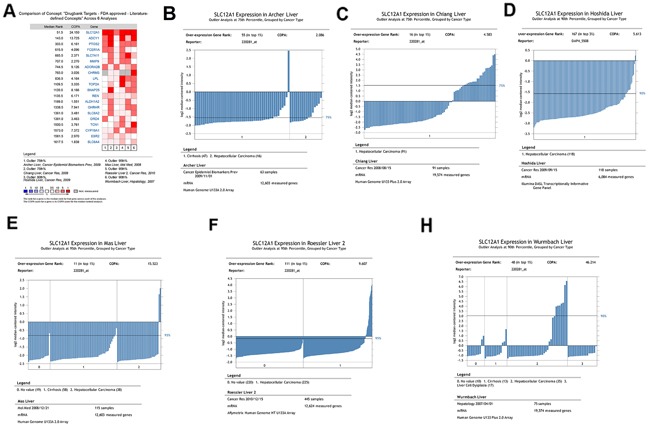
Meta-COPA analysis of HCC gene expression data **A.** Meta-COPA map. Each column in the map represents a HCC gene expression dataset in different papers. Each row indicates a gene. A red cell indicates that the gene was deemed to have an outlier expression profile in the respective dataset for the highest COPA-normalized values for top-scoring meta-outliers across 6 datasets. A total of 20 genes were outliers in a large fraction of datasets. Genes are ranked by their Meta-COPA P values and the median rank. COPA values for outlier genes are shown. **B-H.** The cancer outlier profile analysis of gene SLC12A1 in the datasets of Alcher *et al*., Chiang *et al*., Hoshida *et al*., Mas *et al*., Roessler *et al*. and Wurmbach *et al*.

Firstly, we sought to address the outlier expression pattern of genes in HCC by Meta-COPA analysis. Six datasets [9–14] form Gene Expression Omnibus (GEO) were analyzed using Oncomine online. Results showed that SLC12A1 was significantly upregulated in a subset of HCC samples (Figure 1A). SLC12A1 mRNA was dramatically upregulated in 5%~25% of the samples of single datasets analyzed using COPA (Figure 1B-1H). Also, we analyzed the expressional difference between normal and HCC tissues across three datasets using t-tests [12–14]. The results showed that SLC12A1 expression was the same in normal and tumor groups (Supplementary Figure S1A-S1C). Together, these results indicate that SLC12A1 is consistently upregulated in a small group of liver cancer patients and suggest that SLC12A1 might function as an oncogene in HCC.

### Histone methylation alternation is responsible for SLC12A1 overexpression in Hep3B cells

**Figure 2:**
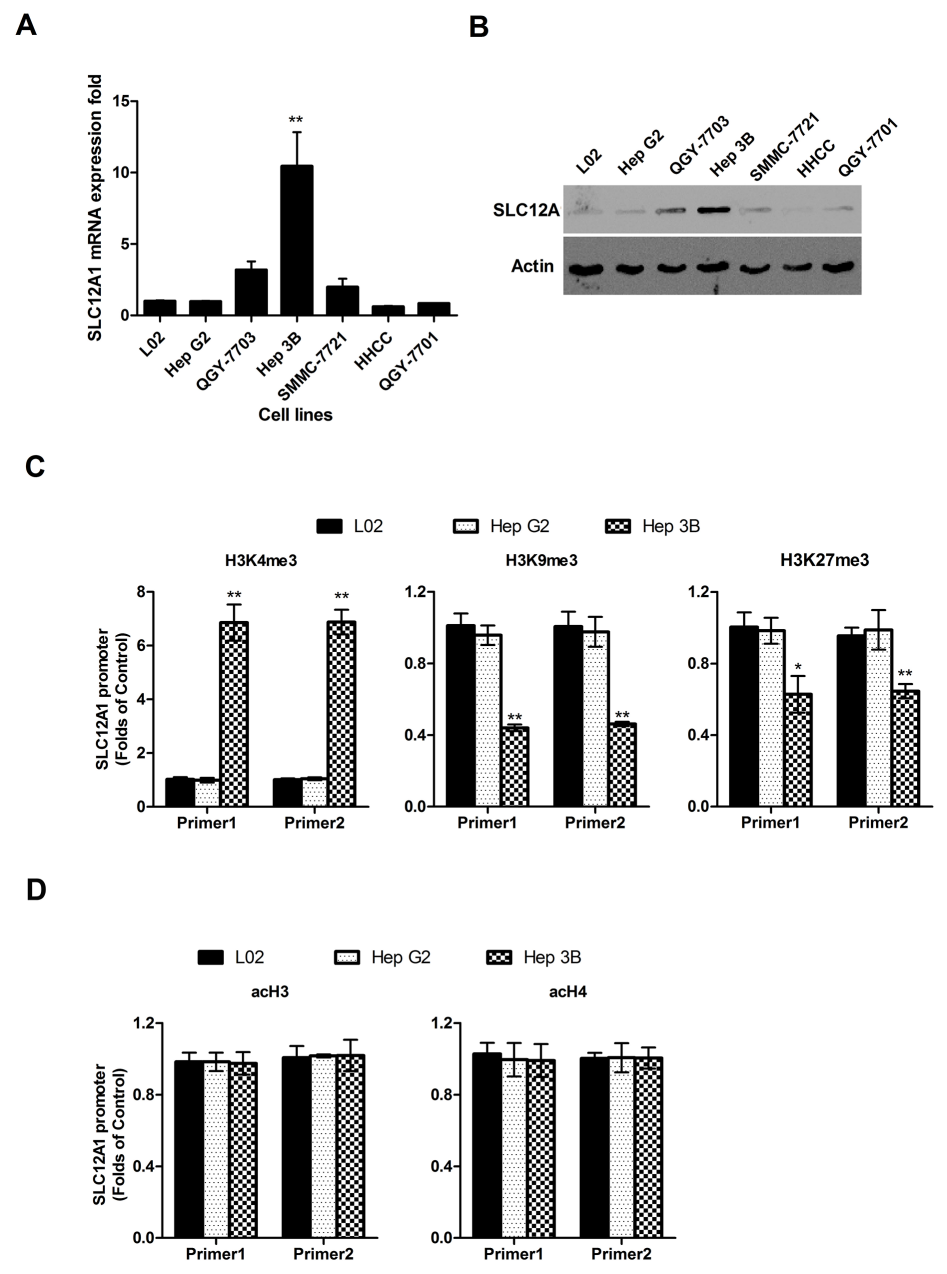
The high expression level of SLC12A1 in Hep 3B cell line **A.** SLC12A1 mRNA expression in one normal liver cell line (L02) and six HCC cell lines (Hep G2, QGY-7703, Hep 3B, SMMC-7721, HHCC, QGY-7701). **B.** The expression of SLC12A1 in one normal liver cell line (L0) and six hepatocellular carcinoma cell lines (HepG2, QGY-7703, Hep3B, SMMC-7721, HHCC, QGY-7701) detected by Western Blot. **C.** ChIP assay for H3K4me3, H3K9me3 and H3K27me3 at the promoter regions of SLC12A1. **D.** ChIP assay for acH3 and acH4 at the promoter regions of SLC12A1 by ChIP assay in L02, HepG2 and Hep3B cell lines. For C. and D., Real-time PCR of GAPDH on the immunoprecipitated DNA fractions was performed as additional internal controls. **, P < 0.01.

Since SLC12A1 was consistently upregulated in a small group of HCC patients, we sought to determine the expression pattern of SLC12A1 in HCC cell lines. Measured by qPCR, the expression of SLC12A1 was increased in Hep3B cells compared to that in hepatic immortalized L02 cells and other five HCC cell lines (HepG2, QGY-7703, SMMC-7721, HHCC, QGY-7701) (Figure 2A). To confirm the robustness of the qPCR results above, we quantified the protein level of SLC12A1 by western blot. Consistent with prior observations, the expression of SLC12A1 was significantly upregulated in Hep3B cells (Figure 2B).

As epigenetic modification regulates gene expression, we tested whether histone modification influenced SLC12A1's expression pattern. The expression level of methylated histone H3 at Lys4 (H3K4), either histone H3 at K9 and K27, and acetylated histones H3 and H4 (acH3, acH4) at the promoter regions of SLC12A1were analyzed in HepG2 and Hep3B cells using ChIP assay (hepatic immortalized L02 cells was utilized as a control). The results indicated upregulation of activation-associated methylated histone H3K4 expressionand downregulation of repression-associated H3K9/H3K27 within the promoter region of SLC12A1 in Hep3B cells (Figure 2C). Meanwhile, the levels of acH3 and acH4 were similar to those of controls (Figure 2D). One representative blot result is shown in Supplementary Figure S2. As Hep3B was isolated from HCC patients, SLC12A1 overexpression in subsets of HCC patients might resulted from histone methylation.

### Inhibition of SCL12A1 inhibits cell proliferation by deactivating the WNK1/ERK5 pathway

**Figure 3:**
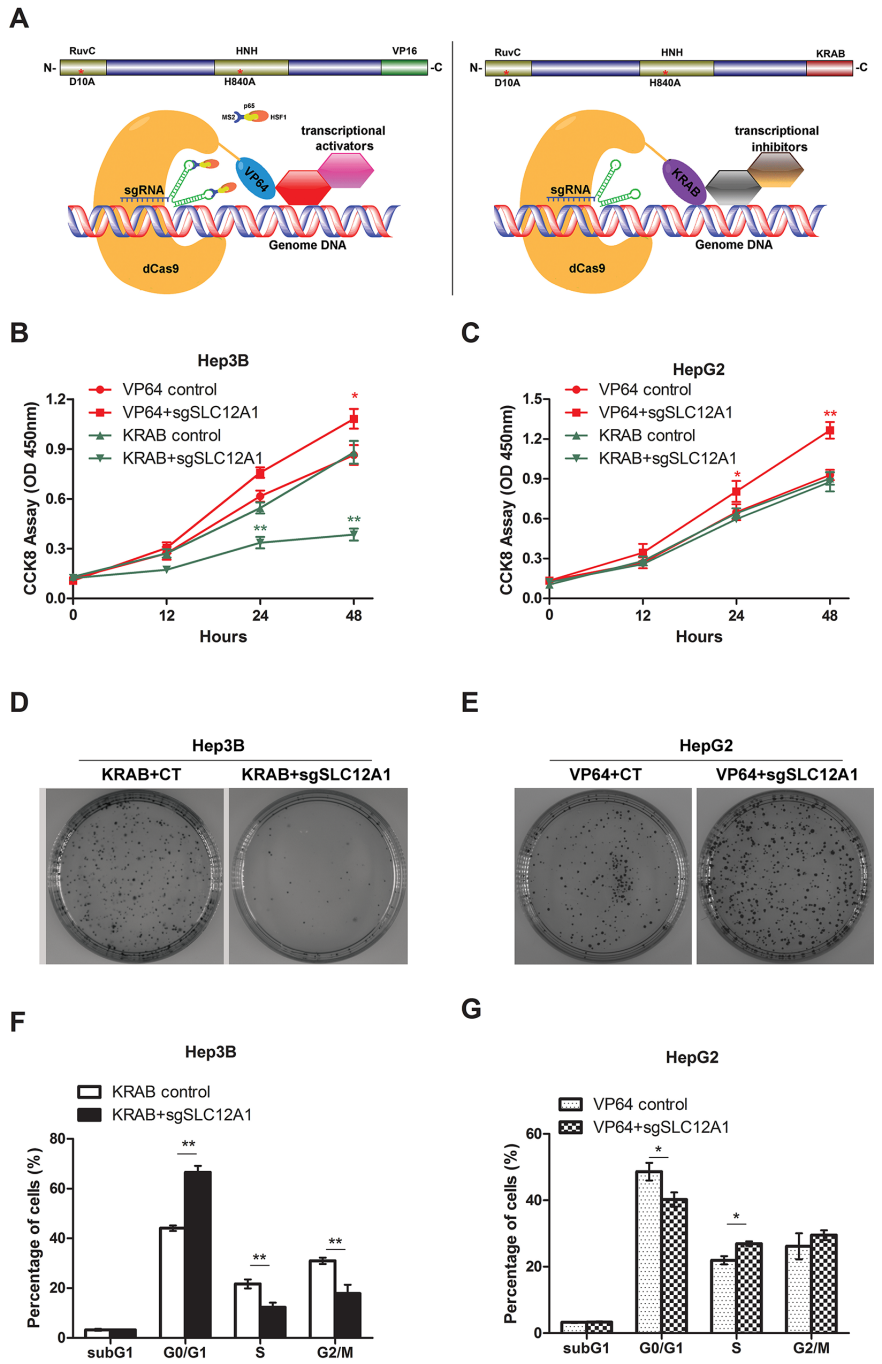
Silencing SLC12A1 inhibits Hep3Bcell proliferation **A-B.** Cell growth of Hep3B or HepG2 cells which were transfected with control RNA and si-SLC12A1 for 0, 24, 48 h was measured by CCK8 assay. **C-D.** Representative wells of the clonogenic growth assay upon knockdown of SLC12A1 (si-SLC12A1) by two independent siRNAs primary cultures in Hep3B or HepG2. Non-targeting siRNA (Ctrl RNA) was used as a control. **E.** Cell cycle distribution of control RNA and si-SLC12A1 transfected Hep3B cells. **F-G.** Expression of the indicated proteins was detected by western blot in control RNA and si-SLC12A1 transfected Hep3B cells at 0, 24, 48 h after transfection. *, P < 0.05; **, P < 0.01.

To further verify the potential role of SLC12A1 as an oncogene in HCC, we used dCas9-VP64 and dCas9-KRAB systems to change the expression pattern of SLC12A1 in SLC12A1-positive cell line Hep3B and SLC12A1-negative cell line HepG2. The dCas9–VP64 system consists of endonuclease-inactive Cas9 (also called dead-Cas9) fused with four transcriptional activators VP16. Also, the dCas9–KRAB system consists of endonuclease-inactive Cas9 fused with transcriptional inhibitor KRAB. The two systems can regulate transcriptional activation or inhibition of particular genes through target gene-specific sgRNAs (Figure 3). The sgRNA was designed as indicated and optimized to find the best combination in HEK293T cells (Supplementary Figure S3). The transcriptional activation and inhibition efficiencies for the selected sgRNAs were measured in Hep3B and HepG2 cells (Supplementary Figure S4).

Next, we assessed the impact of innate activation or inhibition of SLC12A1 on HCC cell proliferation. HCC cell lines HepG2 and Hep3B were infected by lentiviral-packed dCas9-VP64+SAM or dCas9-KRAB with the indicated sgRNAs. Then, cell proliferation was assessed by CCK-8 assay. Knocking-down SLC12A1 expression suppressed cell proliferation in Hep3B cells (Figure 3B), but not in HepG2 cells (Figure 3C). Also, innate activation of SLC12A1 transcription promoted cell growth in both Hep3B and HepG2 (Figure 3A, 3B). The clone formation assay also indicated that SLC12A1 downregulation inhibited proliferation of Hep3B cells (Figure 3D), while SLC12A1 overexpression promoted proliferation of HepG2 cells (Figure 3E). We next investigated the question that why cell proliferation is inhibited by knocking-down SLC12A1. We transfected Hep3B cells with dCas9-KRAB and sgRNAs, and HepG2 cells with dCas9-VP64+SAM and sgRNAs, for 48h. We then stained the cells with propidium iodide (PI), and subjected them to FACS to detect their cell cycle stage. An increased percentage of cells were in G0/G1 phase for transfected Hep3B cells (Figure 3F). On the other hand, we detected a decreased percentage of cells in G0/G1 for transfected HepG2 cells (Figure 3G), indicating that SLC12A1 expression alternation could impact cell cycle progression in G0/G1 phase.

Given the growth inhibition effect of dCas9-KRAB in Hep3B cells, we developed a siRNA against SLC12A1 to investigate the metastasis/invasion ability and EMT process of these cells after SLC12A1 inhibition. As shown in Supplementary Figure S5, the most efficient siRNA was selected by qPCR and western blot. The results indicated that silencing SLC12A1 does not affect invasion/metastasis or EMT in Hep3B cells (Supplementary Figure S6A-S6C).

**Figure 4:**
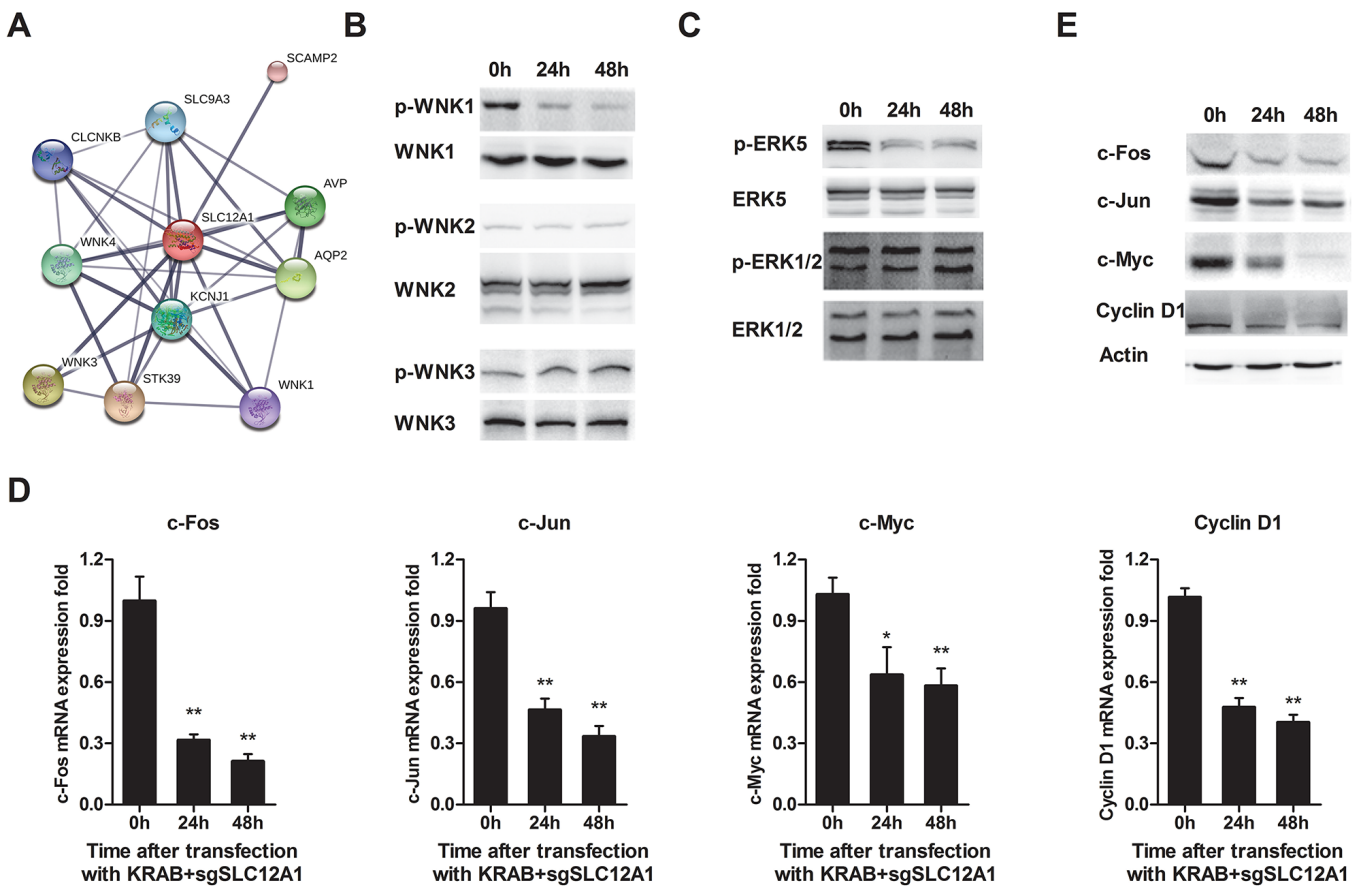
SLC12A1 knock-down in Hep3B impaired WNK1/ERK phosphorylation and blocked expression of downstream genes **A.** Prediction of proteins that interact with SLC12A1 using STRING (http://string-db.org/). The minimum required interaction score was set to 0.75. The thickness of lines between molecules indicates the strength of data support. **B.** Representative western blots for activation of three members of the WNK family involved in the SLC12A1 network. **C.** Representative western blot for ERK1/2 and ERK5 phosphorylation. **D.** The qPCR for c-Fos, c-Jun, c-Myc and Cyclin D1 (*, P < 0.05; **, P < 0.01). **E.** Protein level of genes indicated in C.

We also explored SLC12A1's potential signaling pathways by predicting interaction partners using the online-software STRING. We found three members of theWNK family, a group of serine-threonine protein kinases reported to promote cell proliferation, survival and oncogenesis (Figure 4A). We again used siRNA againstSLC12A1 to investigate its signaling pathways. As shown in Figure 4B, after transfection with siSLC12A1, phosphorylation of WNK1 was inhibited. We then measured activation of downstream protein ERKs by western blot. The results showed that phosphorylation of ERK5 was suppressed. Also, the expression of four genes, including c-Fos, c-Jun, c-Myc and Cyclin D1 (whose transcription directly depends on ERK5) was decreased at both the mRNA level (Figure 4D) and protein levels (Figure 4E).

### SLC12A1 antagonist Bumetanide inhibits SLC12A1-positive HCC cells both *in vitro* and *in vivo*

**Figure 5:**
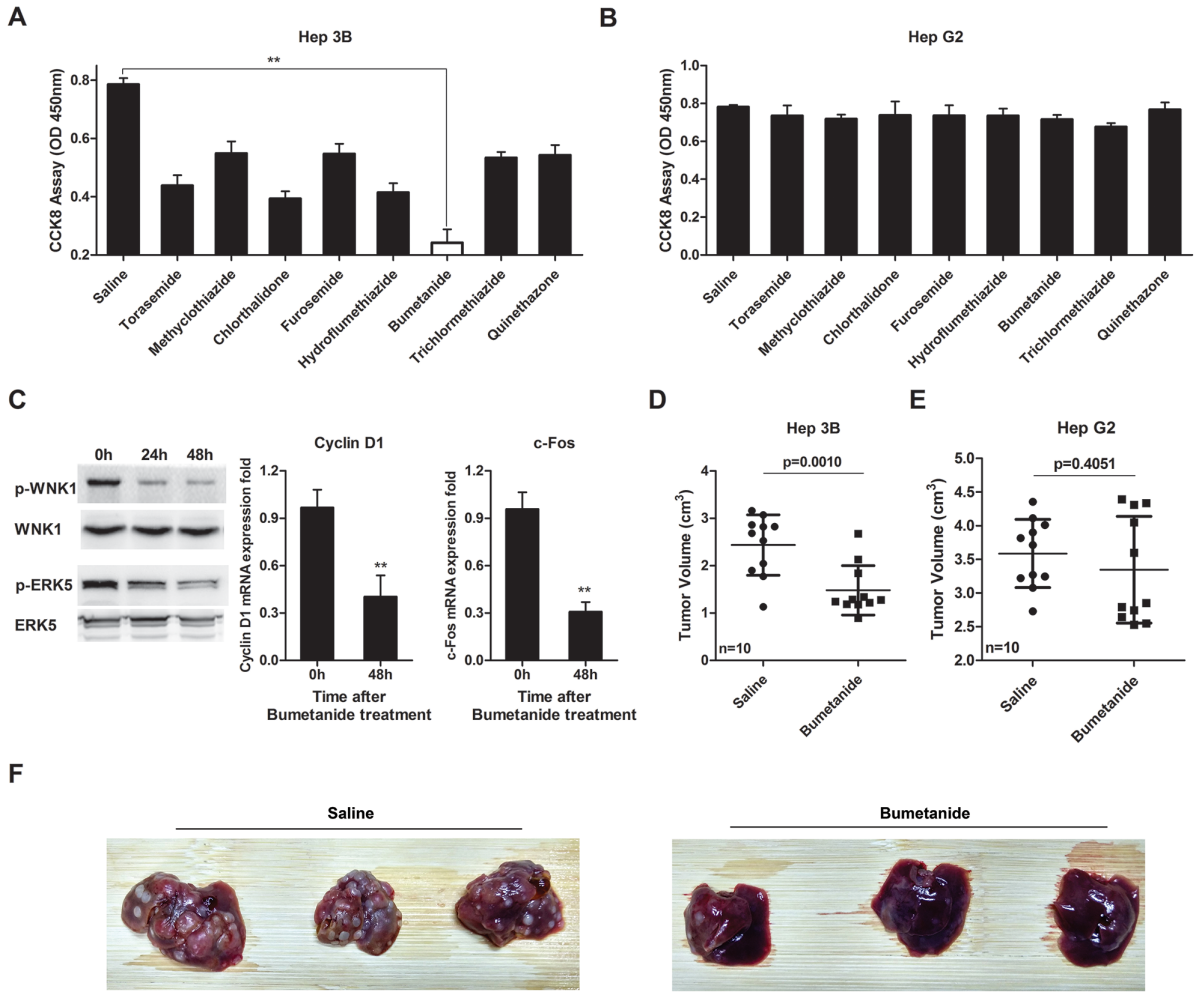
Bumetanide inhibits growth of SLC12A1-postive cells *in vitro* and *in vivo* **A-B.** The effect of 8 diuretics on the cell growth of Hep3B (A) and HepG2 (B) was detected by CCK8 assay (OD 450nm). Cells treated with saline were used as control. **C.** Western blot for activation of WNK1/ERK5 and qPCR for Cyclin D1 and c-Fos expression after Hep3B cells treated with 20ng/mL Bumetanide for the indicated times. **D-E.** Tumor volumes for Hep3B and HepG2 xenografts treated with Bumetanide at a 5mg/kg concentration. T-tests were used to compare the tumor volume between the two groups. **F.** Pictures showing orthotopically-implanted hepatic tumors in livers of mice treated with Bumetanide for 10 days after tumor implantation and sacrificed 3 weeks after finalizing treatment. **, P < 0.01.

Given the oncogene role of SLC12A1 in subsets of HCC, we tested whether the SLC12A1 antagonist Bumetanide had anticancer effects. SLC12A1 transports sodium, potassium, and two chloride ions across the apical membrane, and it was reported as the main target of diuretics [19]. Eight SLC12A1 targeting diuretics were selected in this study, and saline was applied as a control. Among the eight drugs, Bumetanide decreased Hep3B cell proliferation, while having no effect on HepG2 cell proliferation (Figure 5A, 5B). Also, the activation of WNK1/ERK5 and expression of downstream genes were suppressed by Bumetanide in Hep3B cells (Figure 5C). We performed the same experiment in other two SLC12A1-negetive HCC cell lines. Result indicated that SMMC-7721 and HHCC cells were not very sensitive to Bumetanide (Supplementary Figure S7). Also, the proliferation of HepG2 cells transfected with dCas9-VP64+sgRNAs was suppressed by Bumetanide (Supplementary Figure S8A). On the other hand, Hep3B cells transfected with dCas9-KRAB+sgRNAs were not sensitive to Bumetanide (Supplementary Figure S8B). Although Bumetanide is characterized by low toxicity, we nonetheless performed Tunel assay to exclude any potential cytotoxicity. Results indicated that even at1000ng/mL (50x the experimental concentration) would not cause cell damage (Supplementary Figure S9), suggesting that Bumetanide slowed down tumor growth by interfering with the cell cycle rather than by inducing cytotoxicity.

We also assessed the tumor inhibitory effect of Bumetanide *in vivo*. Accordingly, we treated subcutaneous xenograft tumor models with Bumetanide (5mg/kg). After 4 weeks, tumor volumes were measured. Bumetanide treatment decreased tumor volume in Hep3B cells (Figure 5D), but not in HepG2 (Figure 5E). We then applied immunohistochemical analysis to detect SLC12A1 in tumors of xenograft models to test whether SLC12A1 was continuously expressed during tumor formation (Supplementary Figure S10). We also created orthotopically-implanted tumors in mice liver by injecting Hep3B cells into the hepatic portal vein. Bumetanide reduced the size and number of HCC nodules compared with the Saline group (Figure 5F). The above findings indicated that, as an antagonist of SLC12A1, Bumetanide could inhibit liver cancer.

## DISCUSSION

There has been overwhelming evidence that malignant tumors, including hepatocellular carcinoma, are heterogeneous, exhibiting genotypic and phenotypic diversity across patients with the same type of cancer. As a result, tumor subtype characterization is required to improve cancer therapies [15]. Until recently, statistical analysis of expression-microarray data focused on finding genes that were dysregulated in most of the sample of a dataset while heterogeneous expression patterns were deemed to be outliers and received little attention. However, a new analytical method called “Cancer Outlier Profile Analysis” (COPA) [7] was developed to identify genes that are overexpressed in only a small subset of specimens. This method has been integrated into Oncomine [16]. The original outlier detection method normalizes the data such that the median of each expression feature across all samples is 0.0 and the mean absolute deviation is 1.0; then, features are ranked based on their value at the 75th, 90th or 95th percentiles [17]. The utility of COPA and other similar strategies to identify outlier genes from microarray data was demonstrated for several types of cancer [18, 19, 20]. To identify candidate oncogenes, we performed meta-COPA analysis and identified SLC12A1 as the top ranked meta-outlier in HCC.

SLC12A1 (NKCC2) encodes the electroneutral Na^+^-K^+^-Cl^−^ cotransporters, which belong to the Na^+^-dependent subgroup of solute carrier (SLC) 12 family of transporters. SLC12A1 mediates the electroneutral movement of Na^+^ and K^+^, tightly coupled to the movement of Cl^−^ across cell membranes [21–23]. Several studies revealed oncogenic roles for various SLC family members in many types of cancer [24, 25, 26, 27]] and the hypermethylation status of some of them in plasma [28] or their overexpression [29, 30] have been proposed as cancer biomarkers.

To explore the biological function of SLC12A1, we used lentiviral-packed dCas9-VP64 to recruit transcriptional activators that promote gene transcription [31]. Similarly, we used dCas9-KRAB to induce H3K9 trimethylation (H3K9me3) at the promoter and silence gene expression [32]. Unlike traditional protocols, dCas9 mediated transcription regulation is much closer to real physiological regulation. Our results revealed that silencing SLC12A1 or suppressing its signaling with an antagonist inhibited the growth, proliferation and tumorigenesis of Hep3B cells *in vivo* and *in vitro*. Moreover, overexpression of SLC12A1 promoted Hep3B and HepG2 cell proliferation. Therefore, our data suggest that SLC12A1 acts as an oncogene that promotes HCC.

We also studied SLC12A1 signaling by predicting its interaction partners using the STRING database. WNK1, WNK3 and WNK4, which belong to the WNK family of serine-threonine protein kinases [33], were predicted to interact with SLC12A1. WNK proteins have also been reported to promote cancer cell proliferation [34]; therefore, we checked the phosphorylation status of WNKs under SLC12A1 knock-down conditions. Our results indicated that SLC12A1 is a positive regulator of WNK1.

ERK5 is a substrate of WNK1 [35] that promotes cell proliferation and survival. ERK5 is an atypical MAPK that can be activated *in vivo* by a variety of stimulus, including EGF and osmotic shock [36]. Following its activation, ERK5 phosphorylates several targets, especially in the MEF family. Phosphorylation of MEF2C by ERK5 enhances its transcriptional activity, leading to increased c-Jun gene expression [37]. ERK5 also mediates SAP1 phosphorylation, stimulating in turn the transcriptional activity of c-Fos and c-Myc [35]. SGK, a crucial factor, which is closely linked to the G1/S transition of the cell cycle, can be phosphorylated at serine 78 by ERK5 toactivate expression of Cyclin D1, a key proliferation checkpoint [38]. In this study, we provide evidence that SLC12A1 is a positive regulator of WNK1/ERK5 pathway. Therefore, blocking SLC12A1 signaling might inhibit proliferation-related genes like Cyclin D1.

Genetic studies can also provide insights to better inform treatment choices and to develop new therapies [39, 40]. Our findings suggest thatSLC12A1 can be targeted to treat HCC in subpopulations of patients and that SLC12A1 antagonists could function as molecularly targeted therapeutic drugs. Since SLC12A1 induces urine concentration and NaCl reabsorption, it is sensitive to diuretics such as furosemide and Bumetanide [41]. Thus, in our study, eight diuretic drugs were tested for their effect on SLC12A1. We found that one of the eight drugs, Bumetanide, inhibited tumorigenesis and metastasis in a subset of Hep3B-formed HCC *in vitro* and *in vivo*. Because the circumstances of tumor colonization can affect the efficacy of therapeutic agents [45], we used both subcutaneous xenografts and orthotopically-implanted tumor mouse models. SLC12A1 is antagonized by Bumetanide [42, 43]; furthermore, Bumetanide (in combination with photodynamic therapy) significantly inhibited the growth of C6 glioma [44]. Similarly, here we found that Bumetanide acted as an oncogene in a subpopulation of HCC overexpressing SLC12A1.

**Figure 6:**
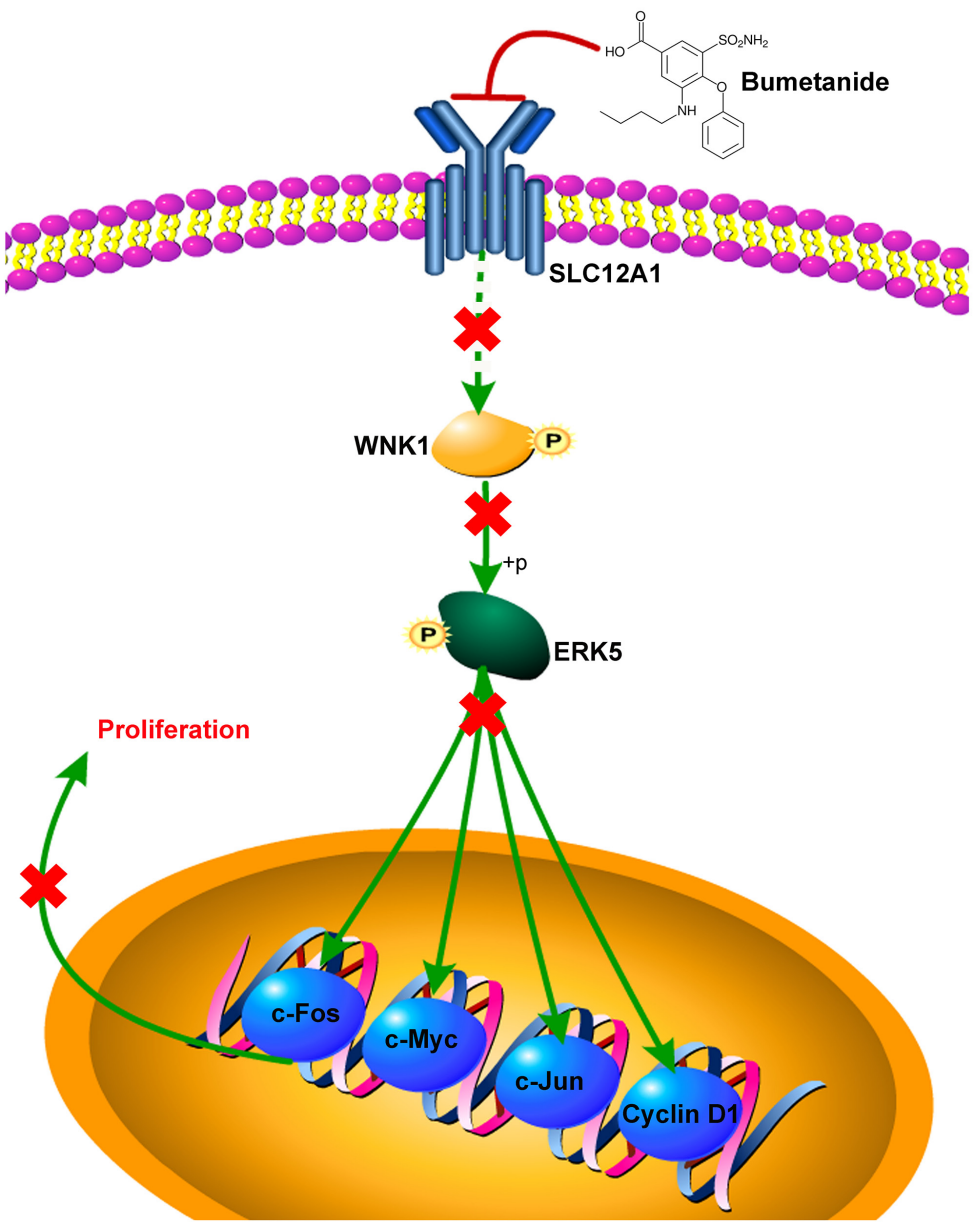
Scheme of proposed SLC12A1 antagonist contributes to inhibition of liver cancer via the WNK1/ERK5 pathway

In summary, our results suggest a novel oncogenic role for SLC12A1 in HCC by promoting cell proliferation through positive regulation of WNK1 in theWNK1/ERK5 pathway. Finally, the SLC12A1 antagonist Bumetanide decreased HCC cell proliferation and inhibited colonization *in vivo* (Figure 6). Therefore, SLC12A1-positive HCC patients might benefit from treatment with selective SLC12A1 antagonists.

## MATERIALS AND METHODS

### Mice and models

BALB/c male nude mice (6 weeks old) were purchased from Joint Ventures Sipper BK Experimental Animal Company (Shanghai, China). Mice were maintained in closed sterile rooms with autoclaved water, fodders and bedding. Infection studies were performed in a different room and each group was housed in a separate isolator. All animal experiments were reviewed and approved by National Institutes of Health Guide for the Care and Use of Laboratory Animals.

To create a subcutaneous tumor xenograft model, 1*10^7^ cells were injected subcutaneously into the flank of nude mice and the xenograft was left to colonize for 10 days. Then, we administrated Bumetanide 5mg/kg via the tail vein daily, the control group mice were injected with the same volume of saline. Tumor tissues were collected 4 weeks after drug administration and the tumor volume was measured. For the orthotopically-implanted tumor model, nude mice inhaled anesthesia with sevoflurane before undergoing surgery in which the abdomen was opened and 5*10^5^ Hep3B cells were injected into the portal vein. Bumetanide 5mg/kg per day were administered intravenously by tail injection for 10 days after tumor implantation. The mice were sacrificed after 3 weeks, and tumor formation in liver was observed.

### Meta-COPA analysis

COPA analysis was performed on 6 HCC gene expression datasets form the GEO database and the data were analyzed using Oncomine online tools (www.oncomine.org). Gene expression values are median-centered, setting each gene's median expression value to zero. Second, the median absolute deviation (MAD) is calculated and normalized to 1 by dividing each gene expression value by its MAD. Of note, the median and MAD were used for transformation as opposed to mean and standard deviation so that outlier expression values do not unduly influence the distribution estimates, and are thus preserved after normalization. Third, the 75th, 90th, and 95th percentiles of the transformed expression values are tabulated for each gene, and then genes are rank-ordered by their percentile scores, providing a prioritized list of outlier profiles. Genes scoring in the top 1% of COPA scores at any of the 3 percentile cutoffs (75th, 90th, and 95th) were deemed outliers in their respective datasets. Meta-outliers were defined as genes deemed outliers in a significant fraction of datasets as assessed by the binomial distribution.

### ChIP assay

The cells were crosslinked and processed according to the Millipore (17-229) Chromatin Immunoprecipitation (ChIP) Assay Kit (Temecula, CA) protocol. Antibodies to H3K4me3 (Millipore 07-473), H3K9me3 (Millipore 05-1242), H3K27me3 (Millipore 07-449), acH3 (Abcam abl0812) and acH4 (Abcam abl5823) were used at 5 mg per immunoprecipitation for the specific immunoprecipitation of respective histone residues. Ten μL of sonicated, preimmunoprecipitated DNA from each sample were used as input controls. ChIP results were analyzed by real-time PCR as described previously [33]. Final results of each sample were normalized to the inputs. Real-time PCR of GAPDH on the immunoprecipitated DNA fractions was performed as additional internal controls [34]. The sequence of SLC12A1 promoter for CHIP was as follows: Primer1: sense-ACCCAACCCCACACCTACTG, antisense-CA CTCTGGGCTGGAACAGTG; Primer 2: sense-ACCC AACCCCACACCTACTG, antisense-TAGCACTCTG GGCTGGAACA.

### Western blot and qPCR

Cells or liver tissues were harvested and lysed with M-PER Protein Extraction Reagent (Pierce, Rockford, IL), supplemented with a protease inhibitor cocktail (Calbiochem). Protein concentrations of the extracts were measured using BCA assay (Pierce) and equalized with the extraction reagent. Equal amounts of the extracts were subjected to SDS-PAGE, transferred onto nitrocellulose membranes, then blotted as described previously [46].

Total RNA was extracted, reverse transcribed, and amplified by qPCR, and related qPCR primers were obtained from PrimeBank (https://pga.mgh.harvard.edu/primerbank/).

### Cell culture and SLC12A1 intervene

HEK293T cells were maintained in RPMI-1640 with 10% FBS. HCC cells were maintained in Dulbecco's modified Eaglemedium (DMEM) with 10% FBS, 1 mM glutamine. As for siRNA transfection, cells were seeded into plates and incubated overnight, and then transfected with RNAs using Oligofectamine™ (Invitrogen CA, USA). The cells were culturedand collected at 0, 24 and 48 h after transfection. Non-targeting siRNA with the following sequence was applied as a control: sense: UUCUCCGAACGUGUCACGUTT, anti-sense: ACGUGACACGUUCGGAGAATT. Three siRNA of SLC12A1 with the following sequences were designed: si-1: sense-CGCGCAAACUGUGUGUUAATT, antisense-UUAACACACAGUUUGCGCGTT; si-2: sense -GCUGGCAAGUUGAACAUUATT, antisense-UAAUG UUCAACUUGCAGCTT; si-3: sense-GUCCAGAGGUUU CUUUAAUTT, antisense-AUUAAAGAAACCUCUGGA CTT.

Lentivirus packed with dCas9-VP64 and MS2-P65-HSF1(SAM) was purchased from Hanbio Biotechnology (Shanghai, China). Lentivirus packed with dCas9-KRAB was purchased from YSY Biotechnology (Nanjing, China). The sgRNA was designed online at GENSCRIPT (http://www.genscript.com/gRNA-database), and had the following the sequence: sgSLC12A1-1: CAACTT AAGTACCACATCAAC; sgSLC12A1-2: CCTCAGGGC TAATGACCTGG; sgSLC12A1-3: GGGGCTTACAG TACTATTCC. The sgRNA was synthesized at Genebioseq (Shanghai, China) and assembled into restriction BsmBI sites of lentiGuide-Puro(Addgene plasmid 52963, a gift from Feng Zhang) following Zhang *et al.*'s protocol [47]. Recombinant plasmids (10μg) were cotransfected into HEK293T with lentiviral packaging vectors pRSV-REV (5 μg), pMDLg/pRRE (10 μg) and vesicular stomatitis virus G glycoprotein (VSVG) expression vector pMD2G (7.5 μg). After three days, the post-transfection culture supernatant was concentrated by ultracentrifugation for 1 h at 30,000 rpm, then resuspended in Hank's buffer. The titer was 5~10^7^IU/mL.

The candidate cells were infected at 10 MOI of dCas9-VP64 or dCas9-KRAB and 5 MOI of each sgRNA in the presence of polybrene (6μg/mL), then cultured for further experiments.

### CCK8 assay

Cell viability was detected by Cell Counting Kit-8 (CCK-8) (Dojindo Laboratories, Kumamoto, Japan). Briefly, the cells transfected with control RNA and si-SLC12A1 were seeded into wells of 96-well plates. Afterward, 10 μl of CCK-8 solution were added to each well, and the 96-well plate was continuously incubated at 37°C for 1 h, then the OD value for each well was read at wavelength 450 nm to determine the cell viability on a microplate reader (Multiskan, Thermo, USA). The assay was repeated three times. The cell viability was calculated as follows: Cell viability (%) = [OD (experiment) − OD (blank)]/ [OD (control) − OD (blank)]*100.

### Clonogenic growth assay

One thousand cells were seeded into each well of a 6-well plate in triplicates and incubated to allow for colony formation for 12 days. The colonies were stained with 0.2% crystal violet in 80% methanol overnight and de-stained after which they were scanned using a Tanon 6200 system.

### Cell cycle analysis

The cell cycle was analyzed by flow cytometry. Briefly, 1×10^6^ cells were harvested and washed in PBS, then fixed in 75% alcohol for 60 min at 4°C. After washing them in cold PBS three times, the cells were resuspended in 1 ml of PBS solution with 40 μg of PI and 100 μg of RNase A (Sigma) for 30 min at 37°C. Samples were then analyzed for DNA content by FACSCalibur.

### TUNEL assays

TUNEL assays were performed according to the manufacturer's protocol using *the In Situ* Cell Death Detection Kit (Beyotime Biotechnology, Shanghai, China). In brief, cells were fixed with 4% paraformaldehyde, washed three times with PBS, and then incubated with 0.1% Triton X-100 and 0.1% sodium citrate for 5 min at 4°C. After two additional washes with PBS, the cells were incubated with the TUNEL reaction mixture for 45min at 37°C in the dark. Finally, the cells were stained with DAPI (100 ng/mL) and then analyzed by fluorescence microscopy.

### Immunohistochemical assay

Tumor sections were deparaffinized and followed by rehydration steps through a graded ethanol series and distilled water and then were treated with 3% H_2_O_2_ in methanol for 30 min to block endogenous peroxidase activity. The sections were rinsed in phosphate-buffered saline (PBS) twice, 5 min each time, and incubated with 10% normal goat serum for 30 min to block non-specific antibody binding. After washing, the samples were incubated with primary rabbit monoclonal antibody SLC12A1 (Abcam ab171747) at 4°C overnight, washed in PBS three times, and then incubated with secondary antibodies (Abcamab98488). Later, the sections were stained with DAB according to the manufacturer's protocols and mounted and photographed using an Olympus Inverted microscope IX73 (Tokyo, Japan).

### Statistical analysis

We performed statistical analyses using an unpaired Student's t-test in SPSS 17.0 for all studies unless otherwise indicated. We considered P < 0.05 to be statistically significant.

## SUPPLEMENTARY MATERIALS FIGURES


