# Supplementary material for: Treatment with an SLC12A1 antagonist inhibits tumorigenesis in a subset of hepatocellular carcinomas
Source: Oncotarget. 2016 Jul 18;7(33):53571–82. doi: 10.18632/oncotarget.10670 (PMC5288206; doi:10.18632/oncotarget.10670)
Supplement: Supplementary file 1 [file oncotarget-07-53571-s001.pdf]

Treatment with an SLC12A1 antagonist inhibits tumorigenesis in a subset of hepatocellular carcinomas

SUPPLEMENTARY FIGURES

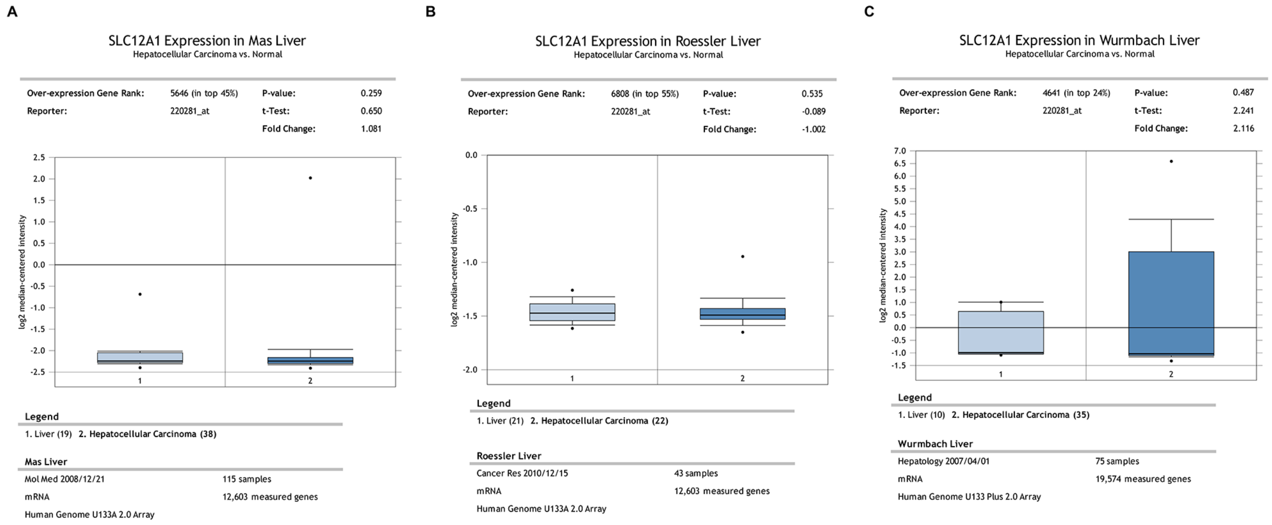

Supplementary Figure S1: T-tests comparing hepatocellular carcinoma vs. normal tissue using the datasets of Mas *et al.*, Roessler *et al.* and Wurmbach *et al.*

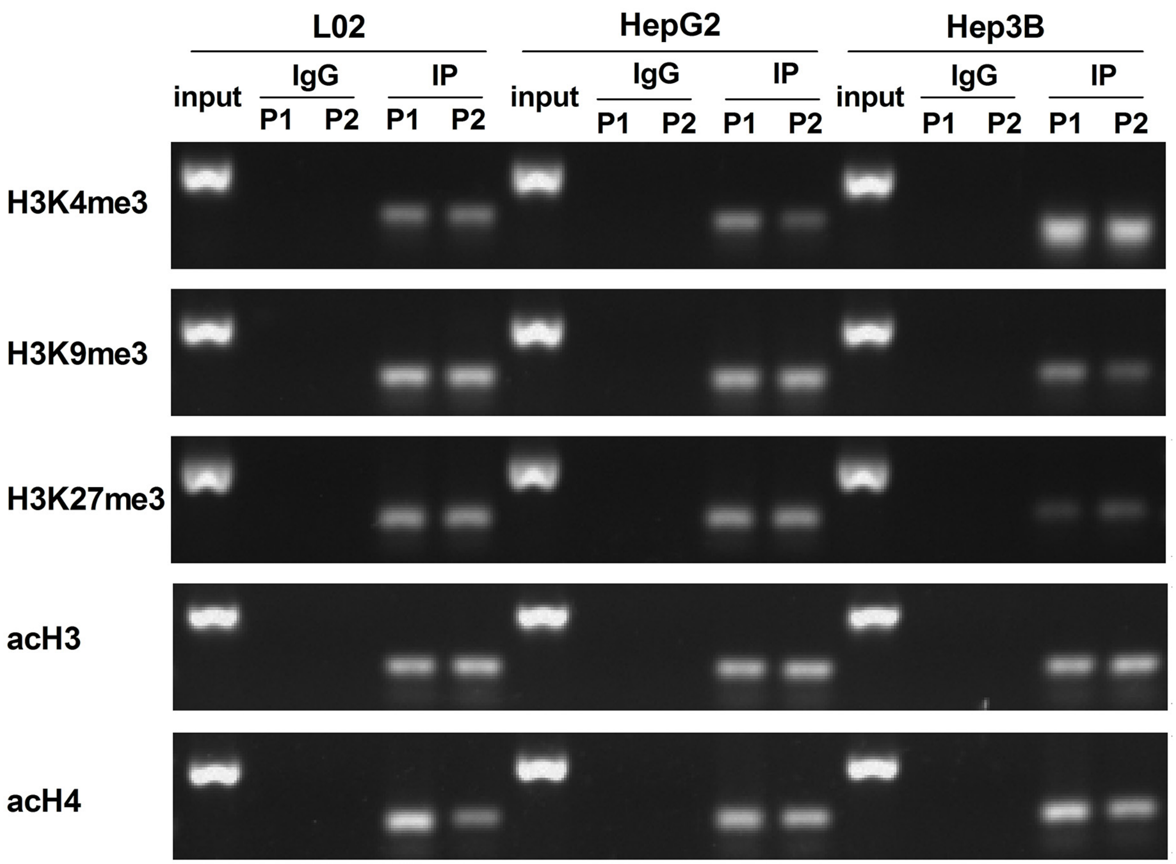

Supplementary Figure S2: Representative blot results of Chip assay for H3K4me3, H3K9me3, H3K27me3, acH3 and acH.4.

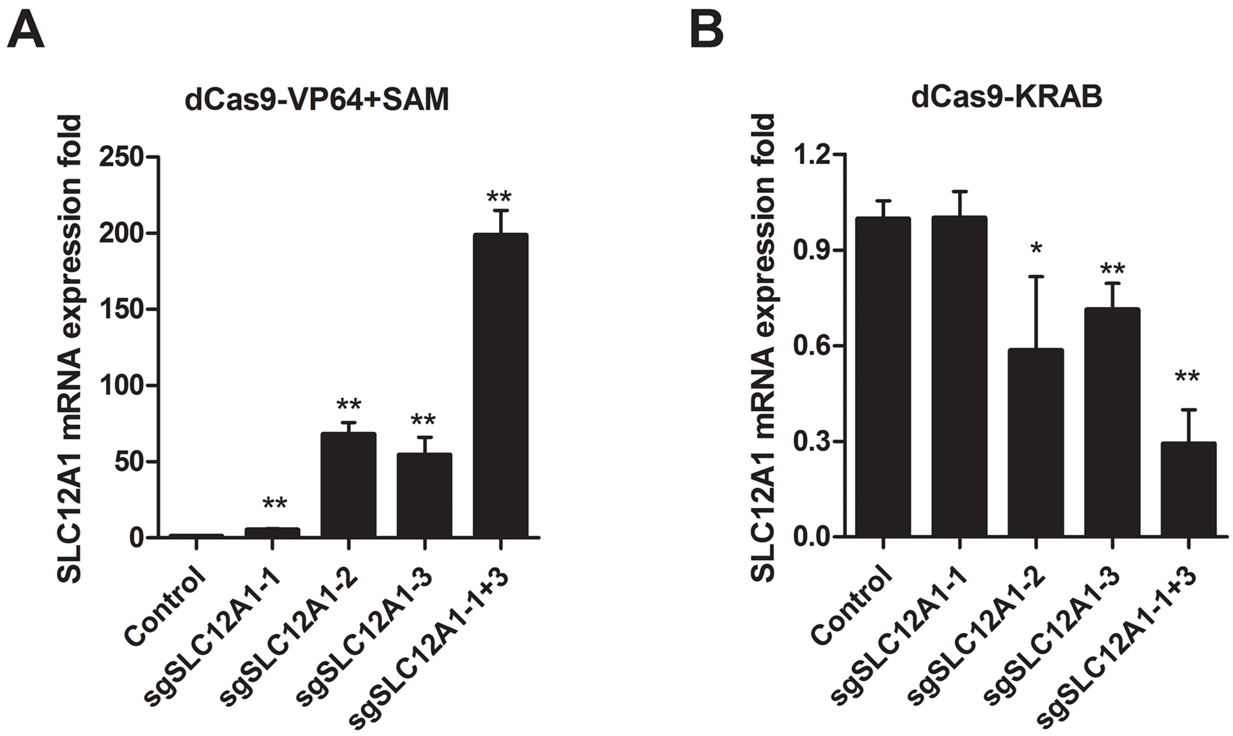

Supplementary Figure S3: Characterization of expression of efficient sgRNAs against SLC12A1 after activation by A. dCas9-VP64 or **B.** suppression by dCas9-KRAB in HEK293T cells.

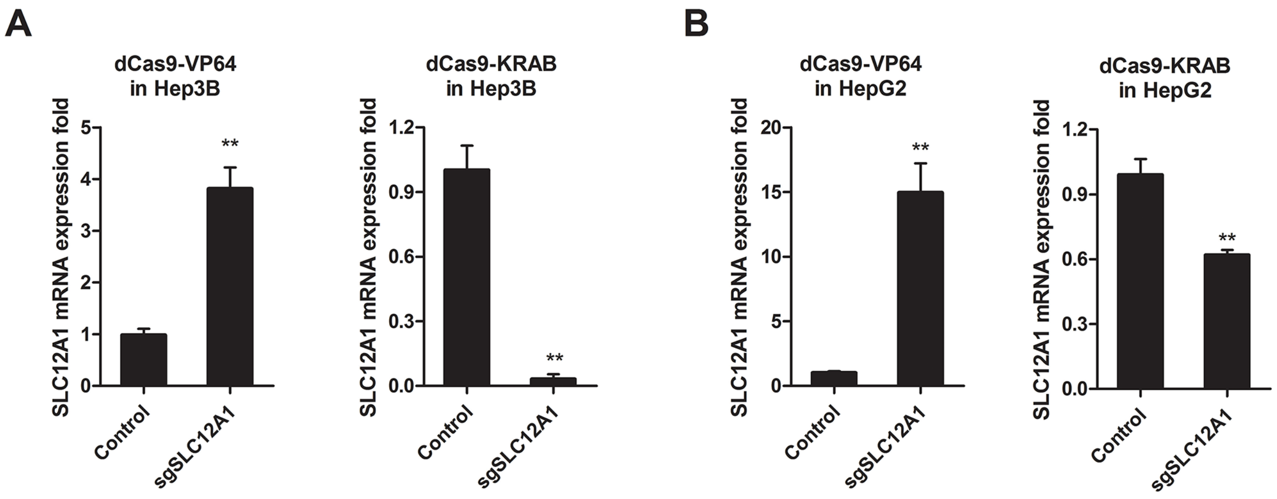

Supplementary Figure S4: Transcriptional activation or suppression efficiencies of sgRNAs in Hep3B and HepG2 cells. The sgSCL12A1 refers to adding 5MOI of sgSLC12A1-1 and 5MOI sgSLC12A1-3 to the system.

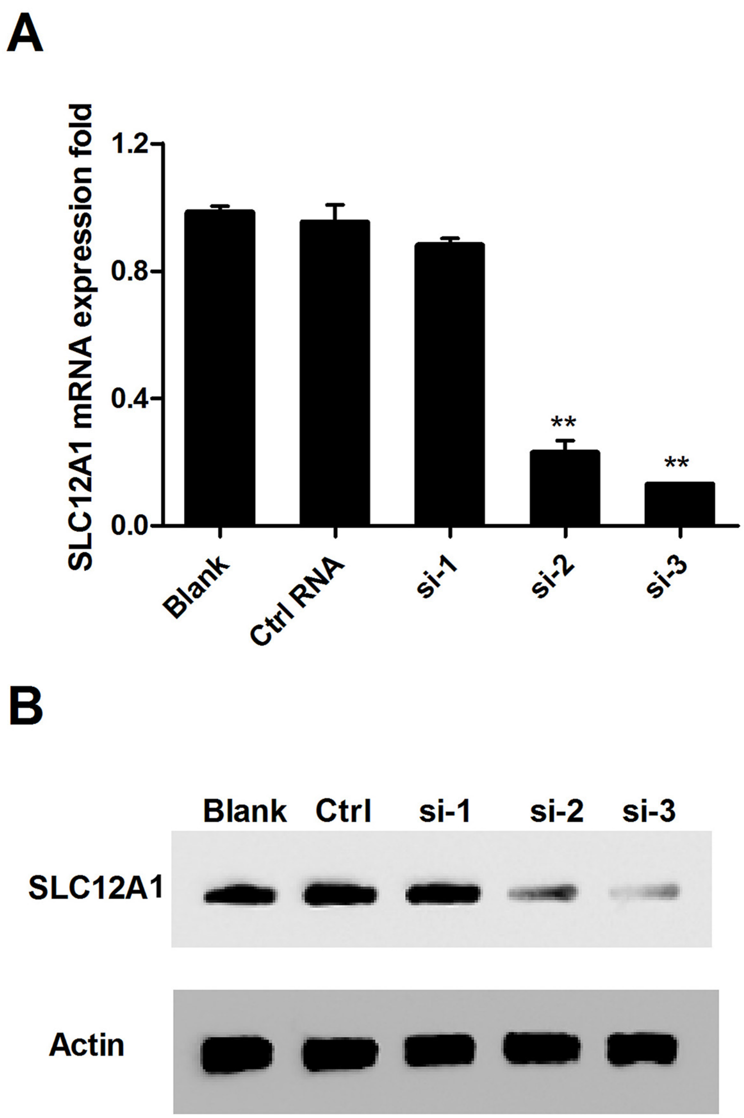

Supplementary Figure S5: mRNA and protein expression levels of SLC12A1 when Hep3B cells were transfected with three si-SLC12A1s and non-targeting si-RNA as a control.

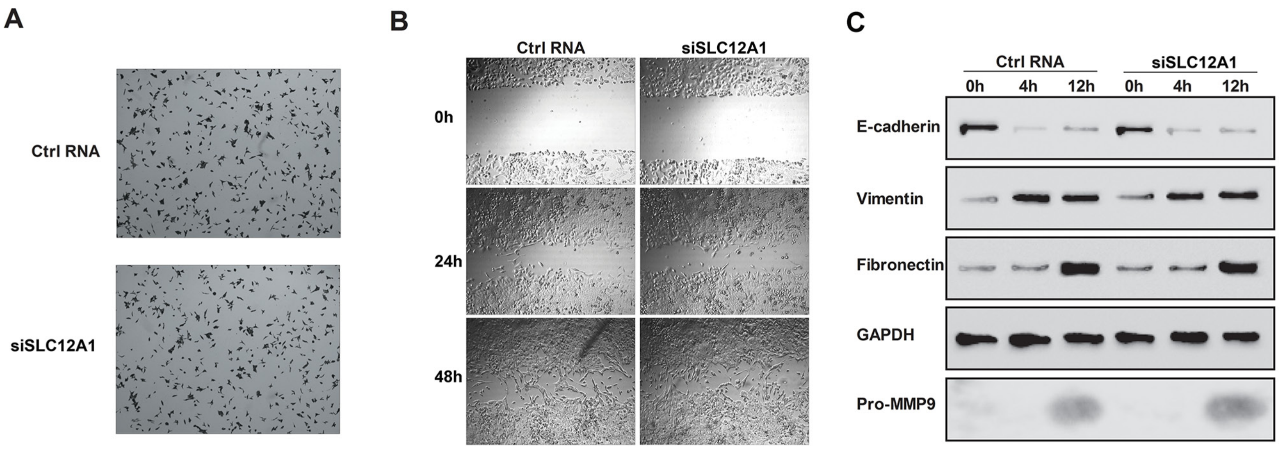

Supplementary Figure S6: Transwell assay, wound scratch healing assay and EMT associated protein detection. Hep3B cells were transfected with siRNA for 24h before performing the experiment. **A.** Transwell assay of Hep3B cells after impairing SLC12A1. **B.** Wound scratch healing assay of Hep3B cell migration after impairing SLC12A1. **C.** Western blot of epithelial markers and mesenchymal markers in Hep3B cells exposed in TGF-beta for the indicated times.

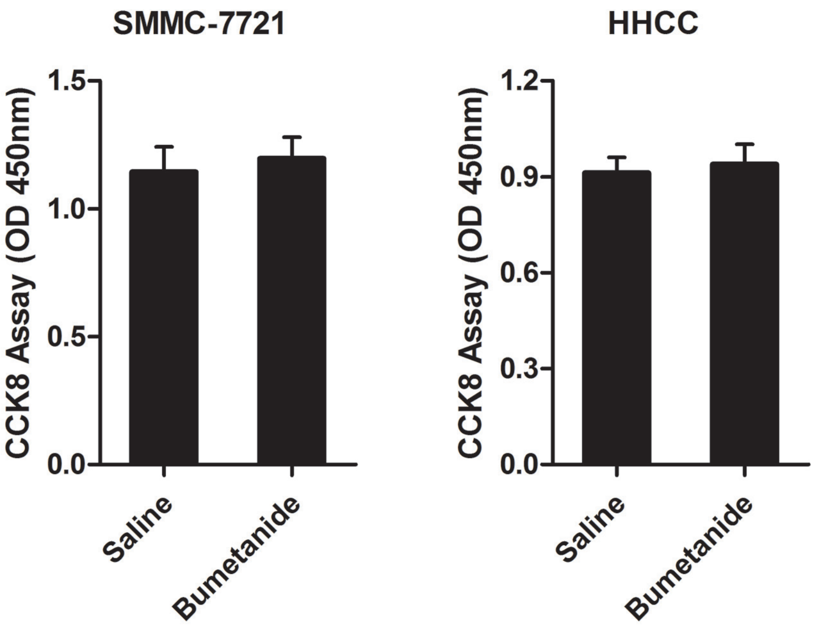

Supplementary Figure S7: CCK8 assay for SMMC-7721 and HHCC cells treated with Bumetanide for the indicated time and with the indicated dose.

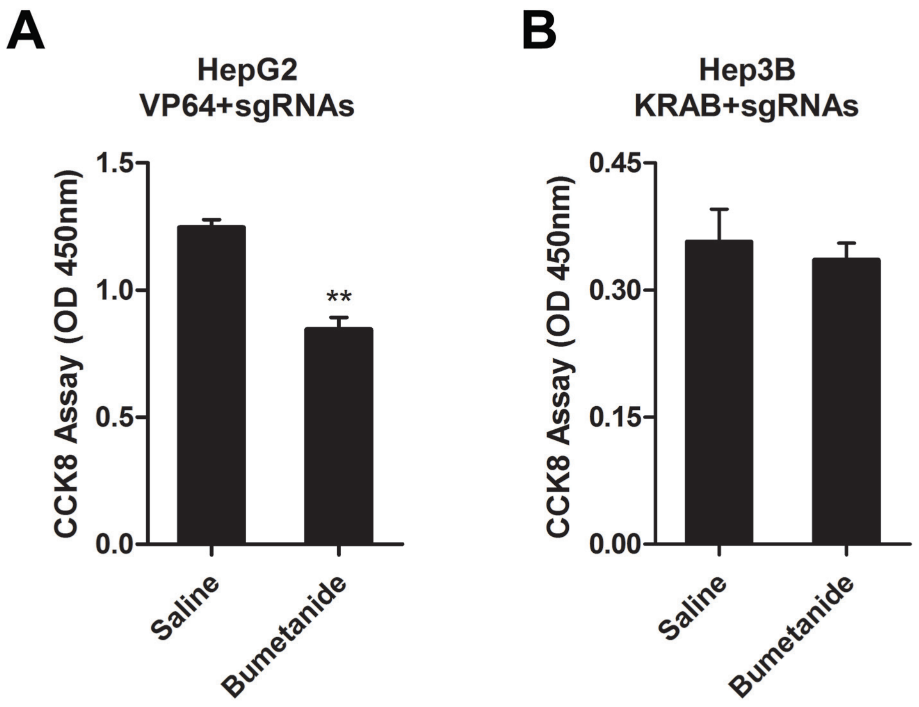

Supplementary Figure S8: SLC12A1 innately activated HepG2 or innately inhibited Hep3B when treated with Bumetanide for the indicated time and with the indicated dose.

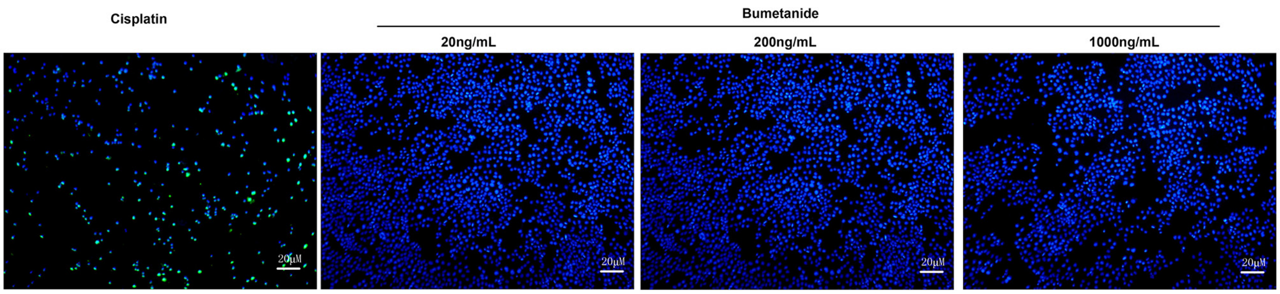

Supplementary Figure S9: Tunel assay for Hep3B cells treated with Bumetanide. Cisplatin with the final concentration of 20ng/mL were used as a positive control, Bumetanide was administrated at the indicated doses. TUNEL (green) indicates apoptotic cells. DAPI (Blue) is used to locate the nuclei of the cells.

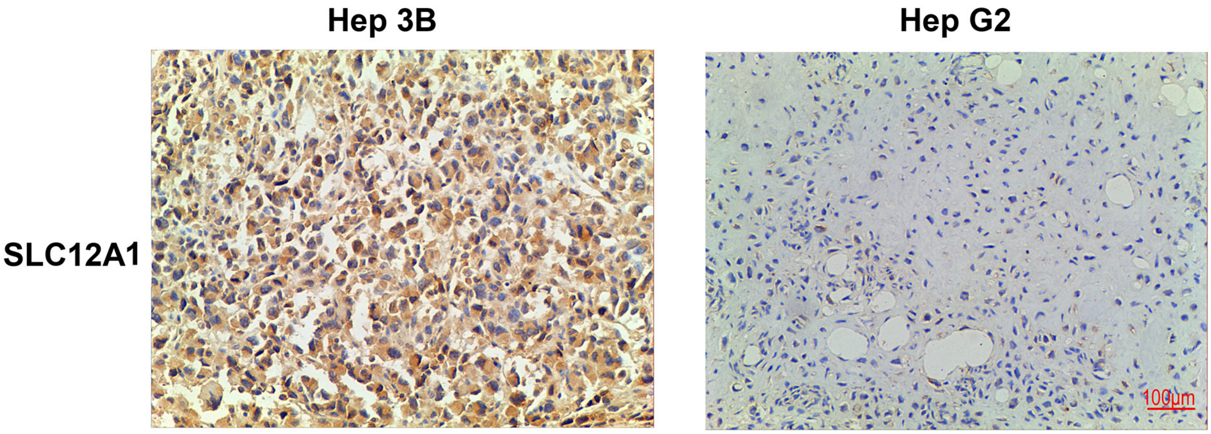

Supplementary Figure S10: Immunohistochemical analysis to detect SLC12A1 in sections from hepatocellular carcinoma formed by Hep3B or HepG2.
